# Structure of tendon causes highly optical anisotropic properties and transport

**DOI:** 10.1117/1.JBO.31.3.035003

**Published:** 2026-03-27

**Authors:** Alexa Nazarian, Steven L. Jacques, Joshua Tam, Richard Rox Anderson, Sandra J. Shefelbine

**Affiliations:** a Massachusetts General Hospital, Wellman Center for Photomedicine, Boston, Massachusetts, United States; b Northeastern University, Department of Bioengineering, Boston, Massachusetts, United States; c University of Washington, Seattle, Washington, United States; d Harvard Medical School, Department of Dermatology, Boston, Massachusetts, United States; e Northeastern University, Department of Mechanical Engineering & Industrial Engineering, Boston, Massachusetts, United States

**Keywords:** tendons, optical properties, optical clearing, scattering, anisotropy

## Abstract

**Significance:**

Tendons are highly anisotropic tissues that exhibit distinct optical properties depending on the direction of light propagation relative to their fiber orientation. Understanding these variations and how to modify them through optical clearing techniques is beneficial for many light-based applications, including photobiomodulation therapy.

**Aim:**

To quantify how tendon optical transport depends on fiber orientation and wavelength, comparing light propagation parallel and perpendicular to the tendon’s long axis, the axis along which collagen fibers are primarily aligned, and to evaluate glycerol-based optical clearing for increasing light penetration.

**Approach:**

The reduced scattering coefficient ($\mu_{s}^{\prime }$) was measured from 400 to 1600 nm in tendon samples oriented parallel to the tendon’s fiber axis (transverse slices) and perpendicular (longitudinal slices) to it. Diffuse reflectance and total transmittance were measured using an integrating sphere and spectrophotometer, and optical coefficients were derived through a theoretical Monte Carlo model. Angular scattering measurements at 633 nm were performed to characterize forward scattering behavior. Power transmission was measured in centimeter-scale tendon sections, and Monte Carlo simulations using the measured optical properties were used to model the transmission experiment and compare orientation-dependent penetration. Measurements were conducted in samples treated with saline or glycerol (10% and 60%). Beam profiler images were also captured to assess light distribution.

**Results:**

Longitudinal tendon sections exhibited consistently higher $\mu_{s}^{\prime }$ values than transverse sections. At 800 nm, $\mu_{s}^{\prime }$ was $9.01\;{\mathrm{mm}}^{-1}$ (longitudinal) versus $0.57\;{\mathrm{mm}}^{-1}$ (transverse). Immersion in 60% glycerol greatly reduced $\mu_{s}^{\prime }$ in both orientations and increased transmitted power. Beam profiler images showed higher intensity in fascicles and fibers relative to the surrounding matrix. This structural pattern diminished with 60% glycerol treatment.

**Conclusions:**

Tendons have strong anisotropic optical properties that favor optical propagation along their fiber axis. Glycerol increases light penetration depths, though further research is needed to confirm safe concentrations for clinical use, as 10% glycerol proved insufficient.

## 1 Introduction

Tendons are a prime example of the relationship between structure and function in biological systems. They are composed of a hierarchical organization of smaller units, with each unit primarily aligned along the tendon’s long axis, the main direction of mechanical stress during function. Fascicles, the largest units, consist of bundles of collagen fibers. These fibers are composed of fibrils, which are crosslinked by hydrophilic chains and formed by collagen molecules arranged in a staggered pattern.^1^^–^^4^

This structural organization and alignment within tendons impart significant mechanical strength necessary for their specialized roles. Positional tendons are adept at transmitting forces from muscle contractions directly to bones, while energy-storing tendons absorb and release energy, helping them handle large forces like springs.^2^^,^^5^ Notably, the anisotropy of tendons extends beyond mechanical properties to their optical characteristics as well. When viewed perpendicular to the fibers, tendons appear white due to strong scattering of light at nearly all visible wavelengths. In contrast, when viewed parallel to the fiber direction, they appear more translucent, suggesting minimal scattering.

This observed optical anisotropy raises the possibility that tendons could facilitate light delivery to deeper tissues along their fiber direction, with potential implications for clinical applications such as photobiomodulation therapy (PBMT). PBMT, which involves the therapeutic use of visible to near-infrared (NIR) light, often faces challenges related to limited light penetration in tissues. Calf muscles like the gastrocnemius and soleus are common PBMT targets^6^^,^^7^ and have shown promising results, such as nearly doubled endurance capacity in mice, measured by time to fatigue on a treadmill.^8^ However, the soleus muscle, which is critical for running performance,^9^ lies deep beneath the gastrocnemius,^10^ making it less accessible to direct light treatment, particularly in humans, where tissue thickness is greater than in mice. Conversely, the Achilles tendon, positioned just 1.6 mm below the skin,^11^ offers a more accessible route for light delivery. By targeting the tendon, light could potentially travel along its length, effectively reaching the soleus muscle more efficiently than direct irradiation through the calf. Tendons may also be the target tissue for PBMT, for example, in the treatment of tendinopathy.^12^

The optical dosimetry for safe and effective PBMT is complex. At least two distinct response pathways have been noted, involving the wavelength-dependent activation of mitochondrial cytochrome c oxidase or the wavelength-dependent activation of transient receptor potential (TRP) channels.^13^^,^^14^ The fluence within tissue for activation is markedly different for each pathway. Therefore, studying and modeling the optical properties of tendons can improve PBMT dosimetry for both muscle and tendons. Monte Carlo simulation tools like MCXLAB,^15^ an open-source software, offer an approach to model light distribution in targeted tissues. These simulations rely on defined optical properties such as the scattering coefficient ($\mu_{s}$), absorption coefficient ($\mu_{a}$), anisotropy factor (g), and refractive index (n), often sourced from the literature. However, a single set of optical properties is commonly used to characterize biological tissues under the assumption of optical homogeneity, which fails to account for the directional dependence of anisotropic tissues like tendons.

Instrumentation and analysis techniques are available to characterize key properties, often combining the scattering coefficient and anisotropy factor into a lumped property known as the reduced scattering coefficient ($\mu_{s}^{\prime }$). The reduced scattering coefficient is defined by the equation:^16^^,^^17^

$$\mu_{s}^{\prime }=\mu_{s}(1-g).$$ (1)

Previous studies have characterized $\mu_{s}^{\prime }$ in tendon over a broad spectral range.^18^^,^^19^ Orientation-dependent reduced scattering coefficients have also been reported; however, these measurements were limited to discrete wavelengths.^20^ The present study quantifies differences in $\mu_{s}^{\prime }$ measured with light incident parallel versus perpendicular to the primary fiber direction across 400 to 1600 nm. Angular scattering measurements at 633 nm were performed to characterize forward scattering behavior in both orientations.

Determining $\mu_{s}^{\prime }$ required knowledge of the absorption coefficient. To approximate $\mu_{a}$, the water absorption spectrum was scaled using the tendon’s estimated water content. This approach avoids the limitations of directly determining absorption via integrating sphere techniques, where losses may be misinterpreted as absorption and where the true absorption of thin tendon slices is only a small fraction of the overall attenuation. Fortunately, in this work, the penetration of light through tendon is primarily governed by scattering, although absorption still contributes to the total attenuation.

Differences in scattering between the two orientations were incorporated directly through the measured $\mu_{s}^{\prime }$ values obtained from each section type. Transverse sections, cut perpendicular to the tendon’s long axis fibers, expose the fibers in cross section, so the incident light propagates parallel to the fibers. Longitudinal sections, cut along the fiber axis, expose fibers running lengthwise, so the incident light propagates perpendicular to the fibers. The Monte Carlo model used to derive the coefficients did not explicitly represent individual fibers. After several scattering events, photons propagate in many directions, and the influence of fiber alignment is captured through bulk transport parameters rather than individual fiber geometry. Modeling oriented fibers could further improve accuracy, but the current approach remains sufficient to highlight the significance of fiber orientation in optical characterizations of tendon.

The reduced scattering coefficients and angular scattering behavior were also evaluated along both measurement orientations after immersion in glycerol, a hyperosmotic agent. This process, known as optical clearing, is used in deep tissue imaging techniques to increase tissue transparency. Prior studies have demonstrated significant optical clearing effects in tendons.^21^^,^^22^ This study assesses the impact of glycerol on the optical anisotropy of tendons and explores its potential to increase light penetration for applications such as PBMT. We assessed the effects of 10% glycerol and 60% glycerol. These concentrations were chosen as experimental conditions rather than proposed clinical treatments, with 10% representing a mild perturbation and 60% included to test the extent of possible clearing effects.

## 2 Materials and Methods

### 2.1 Tendon Samples Preparation

Prefrozen bovine tendons were obtained from a butcher shop and thawed overnight in a refrigerator at 4°C. Three types of samples were prepared. For anisotropy measurements, thin sections with a thickness of 20  μm were prepared. A total of 12 samples were obtained from the same tendon. Six longitudinal slices (cut parallel to the fiber direction) and six transverse slices (cut perpendicular to the fiber direction) were prepared by embedding centimeter-long tendon sections in an optimal cutting temperature (OCT) compound and quickly freezing them using isopentane chilled with liquid nitrogen. These samples were then sliced using a cryotome (CM 1510 S, Leica Biosystems, Nussloch, Germany) and stored at −80°C until measurements were conducted. Each slice was thawed at room temperature and successively immersed in saline, 10% glycerol, and 60% glycerol, with measurements performed after immersion in each solution. Due to the thinness of the samples, the clearing effects were immediate, so overnight immersion was unnecessary. Solutions were carefully applied with a syringe to avoid oversaturating and compromising sample quality.

**Fig. 1:**
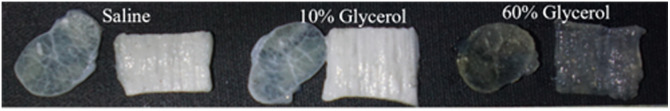
Tendon samples of ∼1.3  cm×2  cm in area and 1.5±0.3  mm thickness. Shown are transverse (left of a pair) and longitudinal (right of a pair) slices that have been immersed in saline, 10% glycerol, and 60% glycerol for 2 days.

For transmission and reflection measurements, 1.5±0.3  mm thick sections were prepared using a custom 3D-printed slicer. This thickness was chosen to ensure sufficient scattering while minimizing light loss from the sample’s edges. A total of 18 slices in the longitudinal orientation and 18 slices in the transverse orientation were obtained from 4 tendons. Six slices of each orientation were immersed in saline, 10% glycerol, and 60% glycerol solutions (Fig. 1), with each group receiving an equal number of samples from the same tendons. The samples were soaked in their respective solutions for two nights at 4°C, and tests were performed the following day.

For the third type of sample, larger tendon sections were immersed in saline, 10% glycerol, or 60% glycerol and refrigerated for two days to ensure thorough absorption. For power transmission measurements, the sections had lengths of 13.8, 13.6, and 10.9 mm for the saline, 10% glycerol, and 60% glycerol, respectively. The diameters of the tendon sections were 13.8, 12.6, and 8.9 mm, respectively. These dimensions were selected so that transmission measurements perpendicular and parallel to the fibers could be compared without length being a significant confounding factor.

### 2.2 Angular Scattering Measurements

**Fig. 2:**
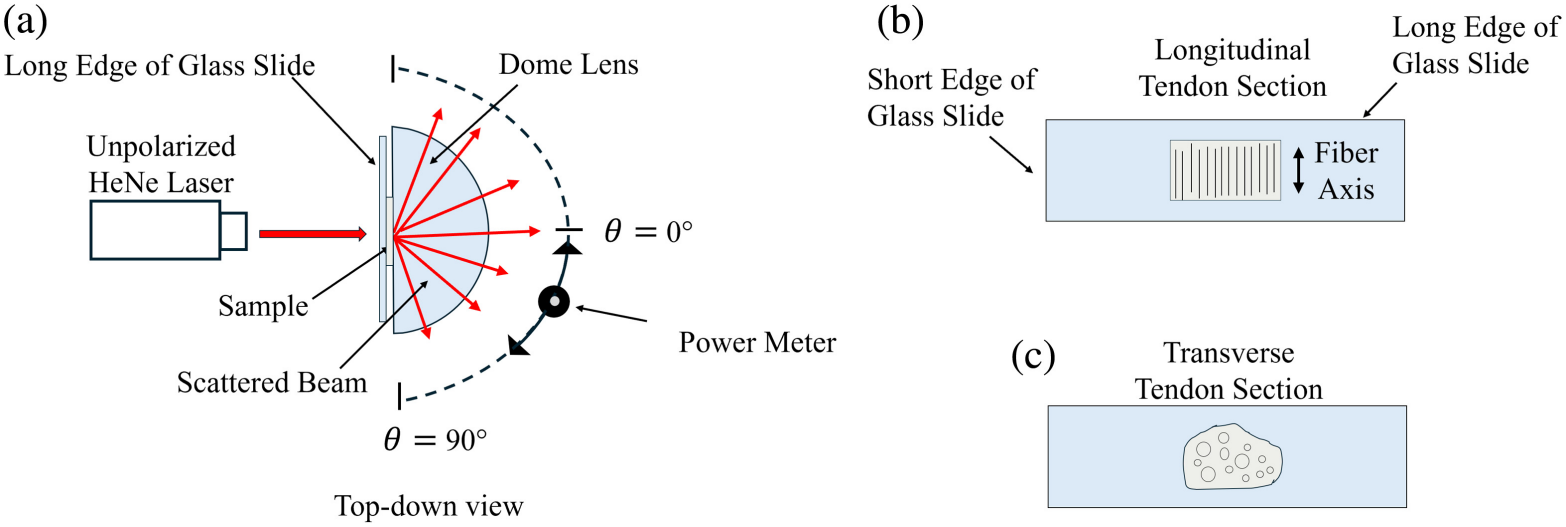
(a) Top-down view of goniometer setup. The He-Ne laser irradiates the sample sandwiched between the dome lens and the glass slide on the center stage. A power meter with a 2.2 mm aperture rotates around the stage to measure the sample’s phase function. The goniometer radius was about 7.8 cm, corresponding to a collection angle of ∼1.6  deg. The polar scattering angle θ is measured in the plane of the page relative to the incident beam direction. The azimuthal angle ϕ lies perpendicular to the page and was not measured. (b) Illustration of a longitudinal tendon section mounted on the face of a glass slide, with the primary fiber axis oriented parallel to the short edge of the slide. (c) Illustration of a transverse tendon section mounted on the face of a glass slide, with fibers oriented into the page. When mounted in the goniometer shown in (a), the incident beam is normal to the slide face (into/out of the page in these views).

A goniometer was used to measure the angular scattering distributions of the samples. A 20-μm-thick tissue sample was positioned in the fixed center of the rotating goniometer stage, and a 633-nm He–Ne laser (Melles Griot, Rochester, New York) with an optical power of 5 mW was aligned so that the incident beam was perpendicular to the cross-sectional area of the tendon (Fig. 2).

The sample was placed between a glass slide and a semicircular prism (dome lens). This configuration eliminates the need for refraction corrections, as the light exits perpendicular to the lens’s tangent upon entering the prism’s center. To achieve precise alignment, the dome lens was secured on a 3D-printed mount and adjusted using a sliding optical base. Vacuum sealant between the glass slide and prism allowed the sample to be slid into position, minimizing mechanical damage during placement.

Optical power was measured using a power meter (PD300, Ophir Optronics, Jerusalem, Israel) fixed on an arm extending from the stage, allowing it to be rotated from −50  deg to +50  deg around the center of the stage, with power measurements taken at each degree. A vernier scale on the stage provided precise angle readings. A 2.2-mm-diameter aperture, held in place by a 3D-printed adapter in front of the power meter sensor, was used to narrow the detection area.

The long edge of the glass slide was parallel to the scattering plane. For longitudinal samples, the fiber axis was oriented parallel to the short edge of the glass slide to align the primary scattering plane (equatorial plane) with the detection plane of the 2D goniometer. When the fiber axis was oriented parallel to the long edge of the glass slide, the dominant scattering occurred perpendicular to the detection plane and was largely missed.

### 2.3 Phase Function Fitting and Effective Anisotropy Estimation

Angular scattering data obtained with the goniometer for both transverse and longitudinal tendon sections were fitted with the Henyey–Greenstein (H–G) phase function, p(θ). The H–G function is expressed with an anisotropy coefficient, g, which quantifies the balance between forward versus backward scattering:

$$p_{\mathrm{HG}}(\theta )=\frac{1}{4\pi }\frac{1-g^{2}}{(1+g-2g\;\cos(\theta ){)}^{3/2}}.$$ (2)

A higher g value corresponds to more forward-scattering behavior, varying from −1 for complete backward scattering, to 0 for balanced forward scattering equal to backward scattering, and +1 for complete forward scattering. However, to more accurately represent the heterogeneity in scattering sources within tendons, including variations from both large collagen fibers and smaller fibrils, the following expressions were used for fitting the data:

$$p_{1}(\theta )=K_{1}p_{\mathrm{HG}}(\theta ,g_{1})\;p_{2}(\theta )=K_{2}p_{\mathrm{HG}}(\theta ,g_{2})\;p_{\text{fit}}(\theta )=\cos(\theta )(p_{1}(\theta )+p_{2}(\theta )).$$ (3)

The function $p_{\mathrm{HG}}(\theta ,g_{1})$ describes the near-on-axis scatter, and $p_{\mathrm{HG}}(\theta ,g_{2})$ describes the more broadly scattered light. Here, $g_{1}$ and $g_{2}$ are simply fitting parameters. They are not the $g_{1}$ and $g_{2}$ parameters associated with the average first and second Legendre polynomials. To align $p_{\mathrm{HG}}(\theta ,g_{1})$ and $p_{\mathrm{HG}}(\theta ,g_{2})$ with the experimental scattering data, the phase functions included scale factors, $K_{1}$ and $K_{2}$, expressed in arbitrary units times steradians (a.u.sr). This scaling was done to convert the theoretical outputs, $p_{\mathrm{HG}}$, measured in inverse steradians (1/sr), to correspond with the fit, $p_{\text{fit}}$, that matched the experimental measurements in arbitrary units (a.u.). In addition, $\left(p_{1}(\theta )+p_{2}(\theta )\right)$ was scaled by a cosine factor to account for the angular Lambertian pattern of escaping flux from a surface. The fitting procedure was performed using experimental data over the angular range −50  deg to +50  deg, corresponding to the measurable range of the goniometer.

A least-squares regression fitting was used to determine the fitting parameters $K_{1}$, $K_{2}$, $g_{1}$, and $g_{2}$ by minimizing the error:

$$\text{error}=\sum (p_{\text{fit}}(\theta )2\pi |\sin(\theta )|d\theta {)}^{2}-(I_{\mathrm{meas}}(\theta )2\pi |\sin(\theta )|d\theta {)}^{2}.$$ (4)

The factor 2π|sin(θ)|dθ accounts for integration over the azimuthal angle under the assumption of rotational symmetry, so that the azimuthally integrated scattering intensity as a function of θ is compared between the experimental data $I_{\mathrm{meas}}(\theta )$ and the fitted model $p_{\text{fit}}(\theta )$.

After fitting, $p_{\text{fit}}(\theta )$ was evaluated over the full angular domain 0 deg to 180 deg (0 to π) by placing the fitted parameters into Eq. (3).

The single-scattering phase function of the tissue was then calculated using Eq. (5) over the full angular domain by removing the Lambertian pattern-of-escape term:

$$p(\theta )=\frac{p_{\text{fit}}(\theta )}{\left|\cos(\theta )\right|}.$$ (5)

Finally, the overall $g_{\mathrm{eff}}$ for the full 360 deg was calculated:

$$g_{\mathrm{eff}}=\frac{\int_{0}^{\pi }\;\cos(\theta )p(\theta )2\pi |\sin(\theta )|d\theta }{\int_{0}^{\pi }p(\theta )2\pi |\sin(\theta )|d\theta }.$$ (6)

This calculation of $g_{\mathrm{eff}}$ was performed for $p_{1}(\theta )$, $p_{2}(\theta )$, and $p_{\text{fit}}(\theta )$ yielding $g_{1,\mathrm{eff}}$, $g_{2,\mathrm{eff}}$, and $g_{\mathrm{eff}}$, respectively [Eqs. (3) and (6)], which assumed the scattering specified within the −50  deg to +50  deg range could be extended to the full 360 deg range using the overall $g_{\mathrm{eff}}$ in the Henyey–Greenstein function.

The validity of treating the 20-μm-thick tendon slices as predominantly single-scattering samples is addressed in the Discussion. For this reason, nomenclature $g_{\mathrm{eff}}$ is used to indicate the effective anisotropy recovered from the measured angular distribution and is treated as the anisotropy parameter in this work.

### 2.4 Diffuse Reflectance and Total Transmittance Measurements

The diffuse reflectance ($R_{d}$) and total transmittance ($T_{t}$) of tendon samples were measured using an internal DRA-2500 integrating sphere connected to a spectrophotometer Cary 5000 UV–Vis–NIR spectrophotometer (Agilent, Santa Clara, California). The integrating sphere included both transmission and reflectance ports, which were larger than the samples (∼12 to 14 mm in width and length). To accommodate this size difference, custom apertures were used to reduce the port size to 8.1 mm.

**Fig. 3:**
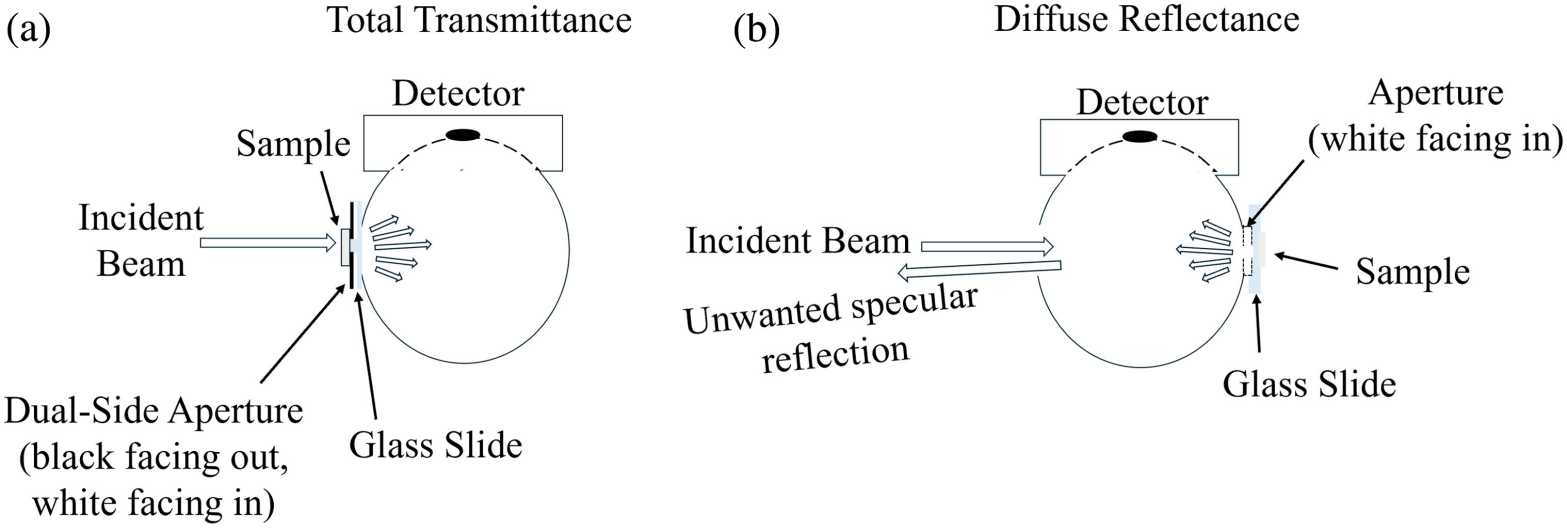
Diagram of integrating sphere setup. (a) Total transmittance measurements. Incident beam passes through the sample first, then through the glass slide and aperture at the transmittance port. The transmitted light is uniformly distributed by multiple reflections inside the integrating sphere and detected by the photodetector. A black aperture blocks external light. (b) Diffuse reflection measurements. The incident beam passes through an aperture and glass slide, strikes the sample at the reflectance port. The reflected light is uniformly distributed within the integrating sphere before detection. Specular reflections exit through the entrance port. The white aperture functions as part of the sphere’s reflective surface.

For total transmittance measurements, an 8.1-mm-diameter aperture was created using light-blocking tape on a glass slide [Fig. 3(a)]. The tape’s black side faced the incoming beam, ensuring that the incoming light passed through the aperture and interacted with the sample first. The white side of the tape, facing the sphere’s interior, helped maximize the uniform scattering of light within the sphere.

For diffuse reflectance measurements, an 8.1-mm-diameter aperture, punched from white cardboard, was taped to the reflectance port with the white side facing the sphere [Fig. 3(b)]. The sample, positioned on a glass slide, was placed directly over the aperture with the glass slide touching the cardboard. To minimize specular reflections from the glass slide, the sphere was configured to allow such reflections to exit through the transmission port.

Calibration was performed using the Cary Scan program. For total transmittance measurements, a baseline for 100% transmittance was established with an empty sample port configuration (glass slide and aperture only), and 0% transmittance was calibrated by blocking the light beam. The software then calculated the transmittance of the samples based on these baselines. For reflectance measurements, a PTFE (polytetrafluoroethylene) white reflectance standard was used as a high reflectance diffuse reference for the 100% reflectance level, and 0% reflectance was calibrated using the aperture and glass slide without the sample. Scans were conducted from 400 to 1600 nm. Some noise around 800 nm was attributed to the integrating sphere switching its detector from NIR to UV-VIS. In addition, the spectrophotometer is polarization-sensitive. To ensure consistency, longitudinal samples were always placed on the glass slide with the same collagen fiber orientation relative to the slide, which remained in a fixed position during all measurements.

### 2.5 Derivation of Reduced Scattering Coefficients

Both diffuse reflectance and total transmittance measurements are affected by the combined effects of absorption and scattering. To precisely isolate and quantify the reduced scattering coefficient ($\mu_{s}^{\prime }$) from these measurements, it is necessary to account for the absorption coefficient ($\mu_{a}$). In this analysis, absorption was modeled based on the dominant contribution of water to tendon absorption over the relevant spectral range. To determine $\mu_{s}^{\prime }$ and approximate $\mu_{a}$, diffuse reflectance and total transmittance were modeled as functions of the optical properties using lookup tables (LUTs) developed from Monte Carlo simulations.^15^ The optical properties were obtained by minimizing the difference between modeled and experimentally measured reflectance and transmittance across the measured spectral range. The optimization was implemented in MATLAB using a trust-region-reflective nonlinear least squares algorithm. The contribution of water was accounted for to approximate the absorption coefficient. This was calculated using the formula^23^:

$$\mu_{a}(\lambda )=W_{t}\mu_{a.\text{water}}(\lambda )\;[\frac{1}{\mathrm{mm}}],$$ (7)

where $W_{t}$ is the water content in the tendon and $\mu_{a.\text{water}}(\lambda )$ is the absorption coefficient of water, characterized by Hale and Querry across a wide spectral range.^24^ While this approximation reflects the high water content of tendon tissue, it is not a direct measurement of tendon absorption but rather a scaled water absorption spectrum.

For the reduced scattering coefficient, a power law with 500 nm as the reference wavelength^23^^,^^25^ was used because water absorption is low at this wavelength, ensuring measurements are predominantly influenced by scattering:

$$\mu_{s.t}^{\prime }(\lambda )=a(\frac{\lambda }{500\;\mathrm{nm}}{)}^{-b}.$$ (8)

Here, a represents the reduced scattering at 500 nm, and b is the scattering power.

To address confounding factors inherent to the integrating sphere setup, such as edge losses at the ports where lateral light spread through the thickness of the sample may go undetected, the experimental data were scaled by a factor K. This scaling aligns the magnitude of the experimental data with the theoretical data so that the sum of reflectance and transmittance nears 100%, as expected in an ideal setup at wavelengths where absorption is minimal (near 500 nm).

The parameters $W_{t},a,b$, and K were adjusted in the iterative least squares algorithm to achieve alignment between experimental and theoretical results, and the upper bound for water content was limited to 65%, consistent with reports that native tendon contains ∼62% water.^26^ The value of $W_{t}$ always reached 65%. In the MCXLAB^15^ Monte Carlo simulations that generated the LUTs, the anisotropy coefficient ($g_{\mathrm{eff}}$), determined using the method described in Sec. 2.3, and the sample thickness, set to 1.5 mm, were entered as simulation inputs for the saline condition, while the g values of the LUTs were 0.91 and 0.98 for the transverse 10% and 60% glycerol samples, respectively, and 0.95 and 0.98 for the longitudinal samples. These g values did not exactly match the goniometer results. To assess sensitivity to the assumed anisotropy, the LUTs were generated over a broad range of g values. At 633 nm, varying g from 0.67 to 0.90 for transverse slices changed the recovered $\mu_{s}^{\prime }$ by 4.8%, whereas varying g from 0.78 to 0.94 for longitudinal slices changed $\mu_{s}^{\prime }$ by 1.6%. This behavior is consistent with the fact that a 1.5-mm tendon slice measured with an integrating sphere lies in a diffuse transport regime, where the measurements are primarily sensitive to $\mu_{s}^{\prime }$ rather than the specific choice of g. The LUT parameter range was adjusted as needed to ensure that all experimentally observed reflectance and transmittance values could be interpolated.

### 2.6 Power Measurements

**Fig. 4:**
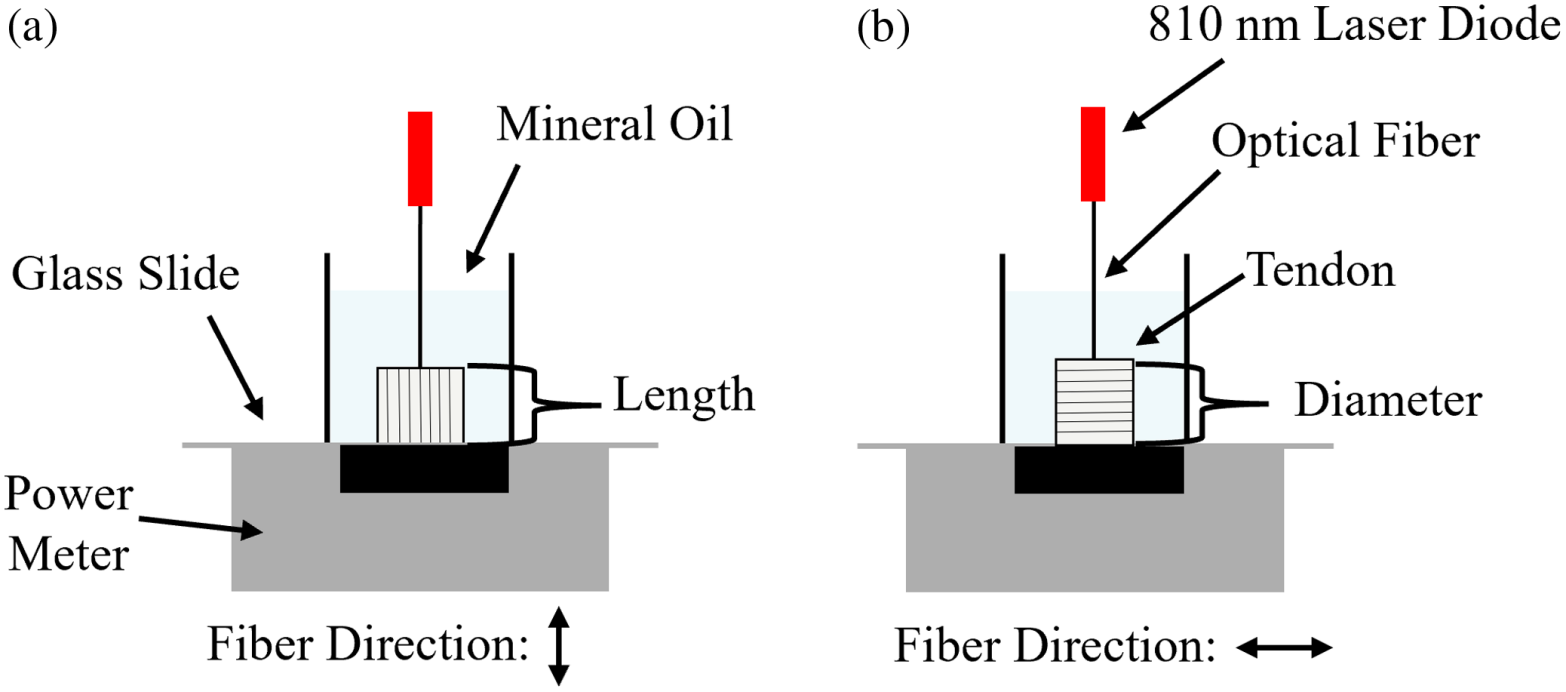
Diagram of power transmission measurement setup. (a) The sample is irradiated via an optical fiber from above contacting the tendon slice, with the beam aligned parallel to the fiber direction, and power is measured from below. The light transmitted through ∼1  cm. (b) The sample is irradiated perpendicular to the fiber direction, and power is measured from below. The length of the sample is trimmed to approximately match its diameter or width, minimizing the influence of tissue thickness as a confounding factor.

Optical power transmitted through the samples along the length of the fiber direction and perpendicular to the fiber direction was measured using a power meter with a sensor (PM100D/S425C, Thorlabs, Newton, New Jersey). The sample was irradiated by an 810-nm laser (Hoya Corporation, Tokyo, Japan), adjusted to output approximately 88 mW at the fiber tip. To minimize inaccuracies from refractive-index mismatches, a sample holder was created by modifying a 50-mL conical tube. The cone part of the tube was removed, and the remaining part was attached to a glass slide. The tube was then filled with mineral oil to better match the refractive index of the tendon, and the tendon sample was inserted into it. Optical power was measured from underneath the glass slide. Unlike the sliced samples used for goniometer and integrating sphere measurements, the tendon here was simply shortened, not sectioned. In Fig. 4(a), light was delivered through a ∼200-μm-diameter optical fiber that touched the tendon surface. The light entered the trimmed face and traveled parallel to the fibers; in Fig. 4(b), the segment was rotated so the beam passed across the tendon’s diameter.

### 2.7 Beam Profiler Images

**Fig. 5:**
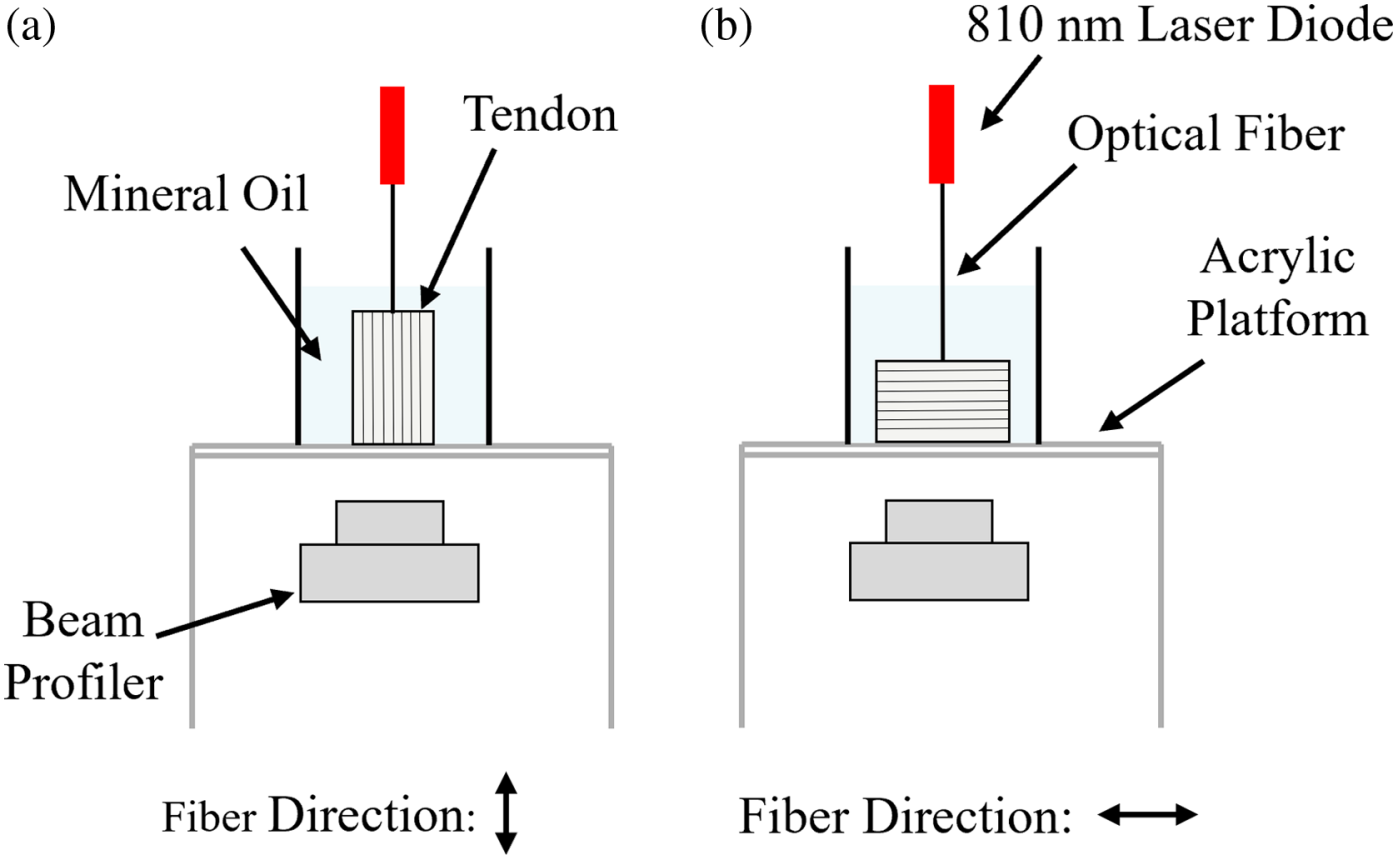
Diagram of beam profiler setup. (a) The sample is irradiated via an optical fiber from above, with the beam aligned parallel to the fiber direction, and imaged from below. (b) The sample is irradiated perpendicular to the fiber direction and imaged from below.

Tendon samples in saline and 60% glycerol were irradiated by an 810-nm laser delivered through an optical fiber. The laser position remained fixed, and the sample was rotated so that the beam entered either parallel to the tendon’s fiber axis [Fig. 5(a)] or perpendicular to it [Fig. 5(b)]. Samples were placed in their sample holders with mineral oil and placed on an acrylic platform. The beam profiler, a high-resolution silicon CCD camera (SP920, Ophir Optronics, Jerusalem, Israel) with a 50-mm CCTV lens, was used to capture images from the opposite side. By adjusting spacers between the lens and camera, the beam image was magnified on the CCD sensor. The camera, mounted on a vertical post, was focused on the bottom surface of the tendon.

## 3 Results

### 3.1 Angular Scattering Distribution

**Fig. 6:**
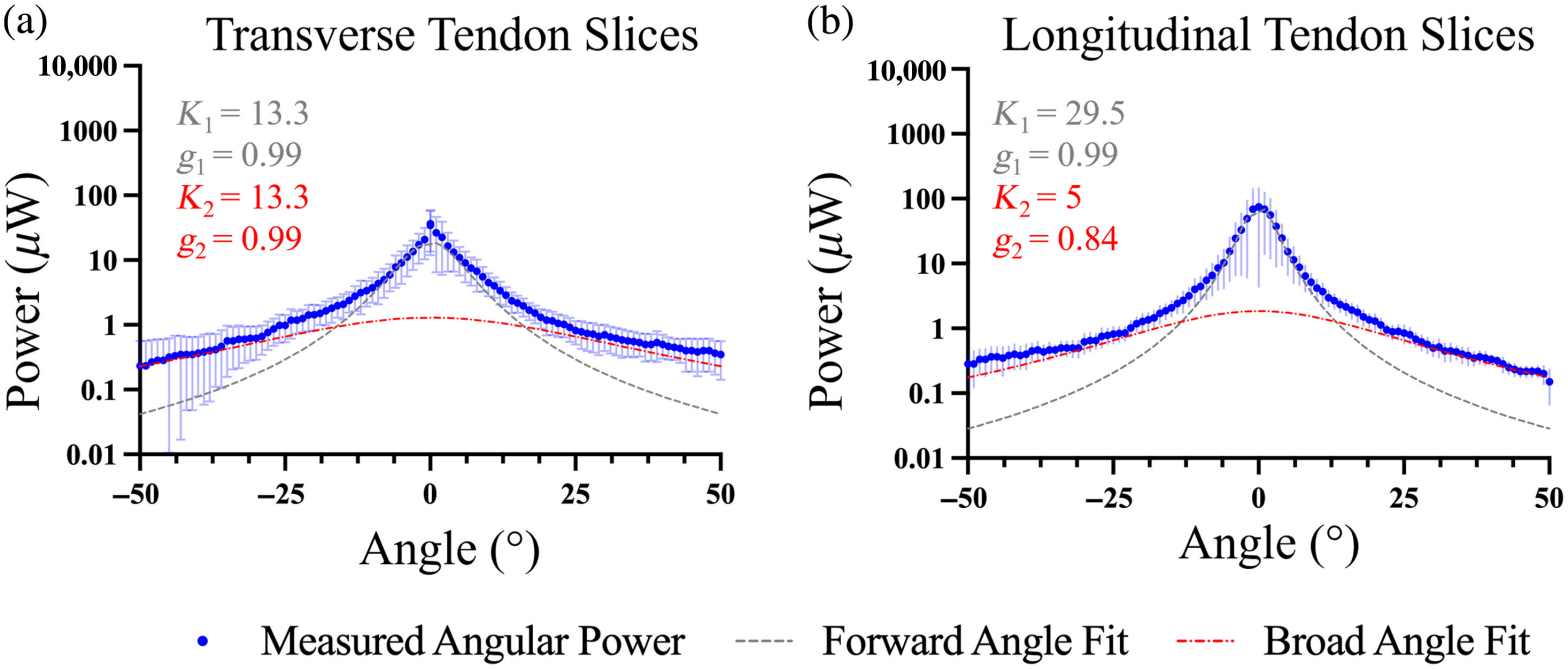
Goniometric measurements of 20-μm-thick tendon samples at 633 nm wavelength for (a) transverse and (b) longitudinal slices soaked in saline. $K_{1}$ and $g_{1}$ correspond to the forward angle fit. $K_{2}$ and $g_{2}$ correspond to the broad angle fit.

Measurements of the angular scattering distribution for transverse and longitudinal tendon slices in saline are shown in Figs. 6(a) and 6(b). The fitted parameters ($K_{1}$, $K_{2}$, $g_{1}$, and $g_{2}$) are displayed on the plots, and the resulting $g_{1,\mathrm{eff}}$, $g_{2,\mathrm{eff}}$, and $g_{\mathrm{eff}}$ values are shown in Table 1.

**Table 1:** Anisotropy of scatter for tendon soaked in saline when light propagates perpendicular versus parallel to the axes of the collagen fibers. Values were obtained from fits to the averaged angular scattering distributions (Fig. 6); Fig. 7 shows mean ± SD across specimens (n=6).

Tendon slice	Light trajectory	$g_{1,\mathrm{eff}}$	$g_{2,\mathrm{eff}}$	$g_{\mathrm{eff}}$
Transverse slices	Light parallel to fibers	0.90	0.57	0.70
Longitudinal slices	Light perpendicular to fibers	0.94	0.65	0.80

As shown in Table 1, both transverse and longitudinal tendon slices exhibited high forward scattering, reflected in the $g_{1,\mathrm{eff}}$ and $g_{2,\mathrm{eff}}$ values for each orientation. Although the overall effective anisotropy $g_{\mathrm{eff}}$ was lower for the transverse slices (0.70) compared to the longitudinal slices (0.80). This difference reflects the relative weighting of the two scattering lobes: in the transverse slices, the broader lobe ($g_{2,\mathrm{eff}}$) contributes more to the total power relative to the narrow forward lobe ($g_{1,\mathrm{eff}}$). In contrast, the longitudinal slices are dominated by a narrower, more forward-directed component, resulting in a higher $g_{\mathrm{eff}}$.

**Fig. 7:**
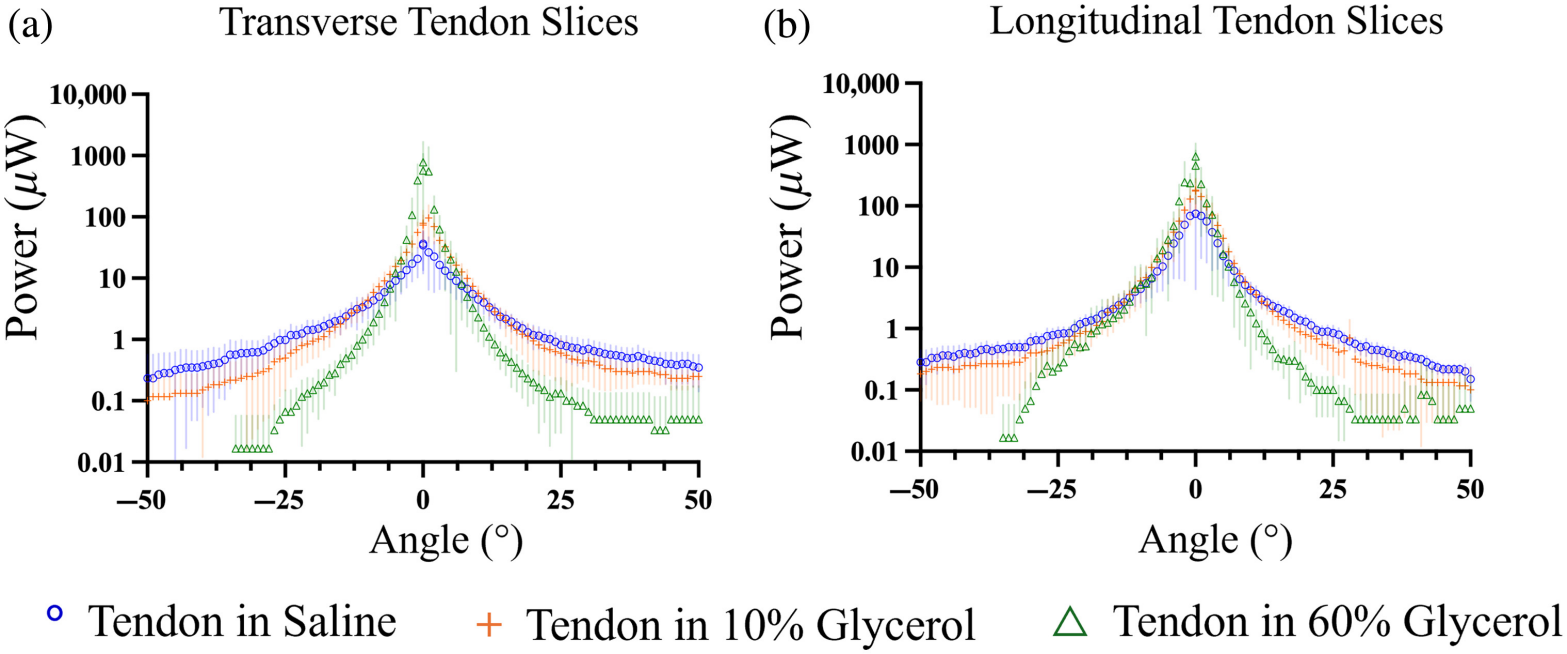
Goniometer data for tendon slices in saline, 10% glycerol, and 60% glycerol. Data points represent mean values (n=6) and standard deviations are indicated by the shaded areas. (a) Transverse slices, where light is incident parallel to the axes of collagen fibers. (b) Longitudinal slices, where light is incident perpendicular to the axes of collagen fibers.

Figure 7 shows the angular scattering distributions of tendon slices in saline, 10% glycerol, and 60% glycerol. For both transverse and longitudinal tendon slices, the scattering profiles became progressively peaked with increasing glycerol concentration, indicating greater forward-directed scattering. Broad-angle scattering decreased correspondingly and was largely absent at 60% glycerol.

A couple of observations from the experimental setup should be considered when interpreting the results. Minor power detections between −35  deg and +8  deg without a sample suggested that the laser beam may not have been fully collimated. The thinness of the samples could affect measurements near 0 deg, as holes might allow light to pass through without scattering. Furthermore, a slight increase in power, approximately 0.1  μW, observed at wide angles in the transverse samples, appeared as slightly higher data points on the right side of the axis at the tail end compared to the left. This asymmetry could be due to stray light or to measurements falling below the sensitivity threshold of the power meter, both of which could affect the accuracy of the readings.

### 3.2 Diffuse Reflectance and Total Transmittance Measurements

**Fig. 8:**
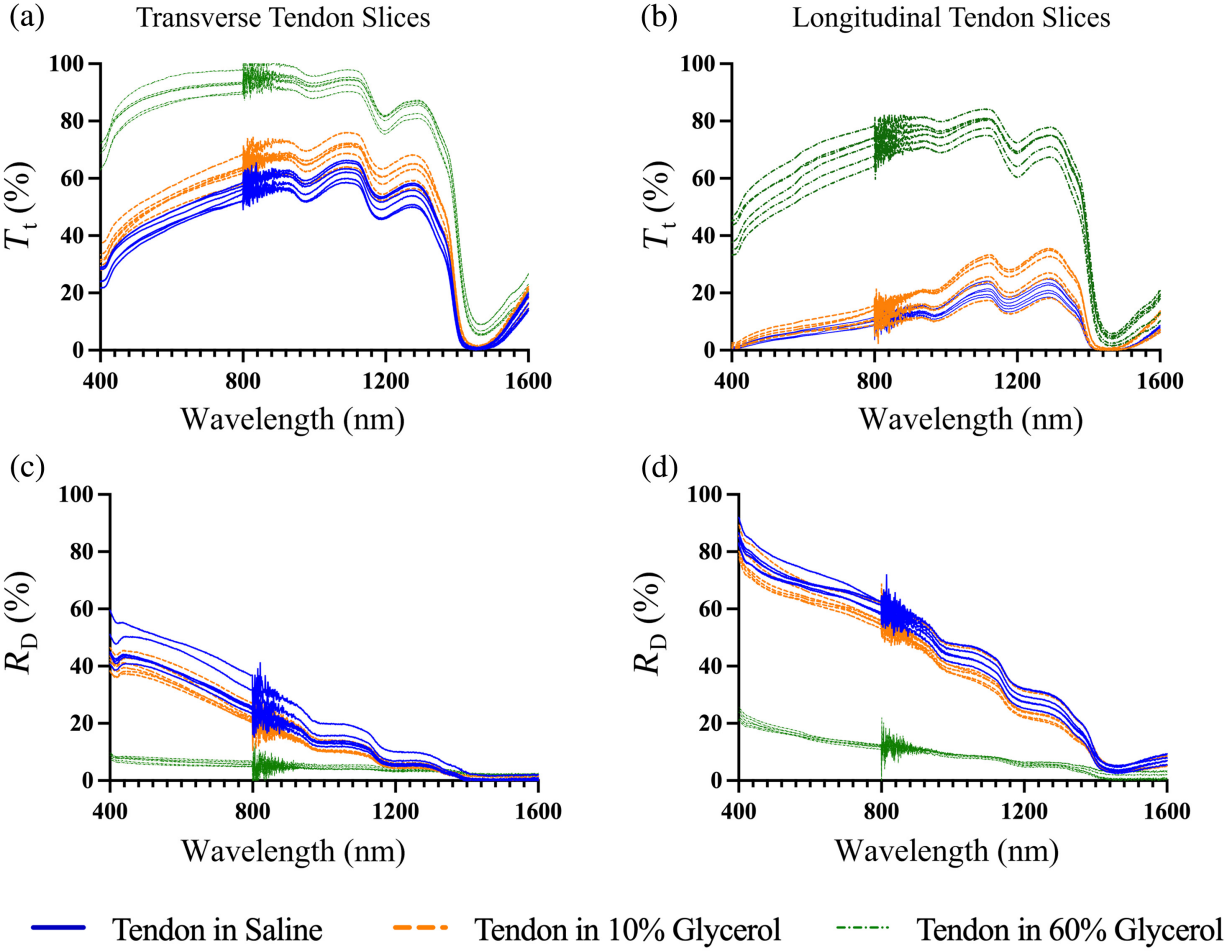
Unscaled total transmittance ($T_{t}$) and diffuse reflectance ($R_{d}$) data of tendon slices (1.5±0.3  mm thick). Total transmittance is shown in (a) and (b), and diffuse reflectance in (c) and (d). (a) and (c) correspond to transverse slices, and (b) and (d) correspond to longitudinal slices. The noise around 800 nm was attributed to the integrating sphere switching its detector from NIR to UV-VIS.

Total transmittance and diffuse reflectance measurements were conducted on three groups of six transverse slices and three groups of six longitudinal slices, with each group subjected to different solutions: saline, 10% glycerol, and 60% glycerol. Total transmittance and diffuse reflectance spectra were acquired across each group and analyzed on a group-averaged basis, rather than as paired measurements from individual slices. The results are presented in Fig. 8.

The noticeable dips in the measurements, such as at 1450 nm and smaller ones at 970 and 1200 nm, align with water absorption peaks, reflecting the high water content in the tendon. In addition, a minor hemoprotein peak is observed at 415 nm due to the presence of small amounts of blood in the *ex vivo* tendon. Despite these characteristics, clear trends can be observed. Transmittance increased from saline to 10% glycerol and more significantly to 60% glycerol for both longitudinal and transverse slices and decreased toward shorter wavelengths. Overall, the total transmittance of transverse slices was greater than that of longitudinal slices for all sample groups. In addition, the diffuse reflectance of longitudinal slices was greater than that of transverse slices. For the samples subjected to 60% glycerol, longitudinal and transverse slices exhibited nearly 100% transmittance and close to 0% diffuse reflectance. This observation is consistent with the visual appearance of the samples in Fig. 1, where they appear clear. At wavelengths with minimal water absorption, such as 500 nm for transverse slices in the saline group, the sum of the total transmittance and diffuse reflectance was 84%. For longitudinal tendon slices, the sum was 77%, suggesting some light loss within the setup, potentially due to lateral light spreading through the sides of the tissue sample before entering the integrating sphere.

**Fig. 9:**
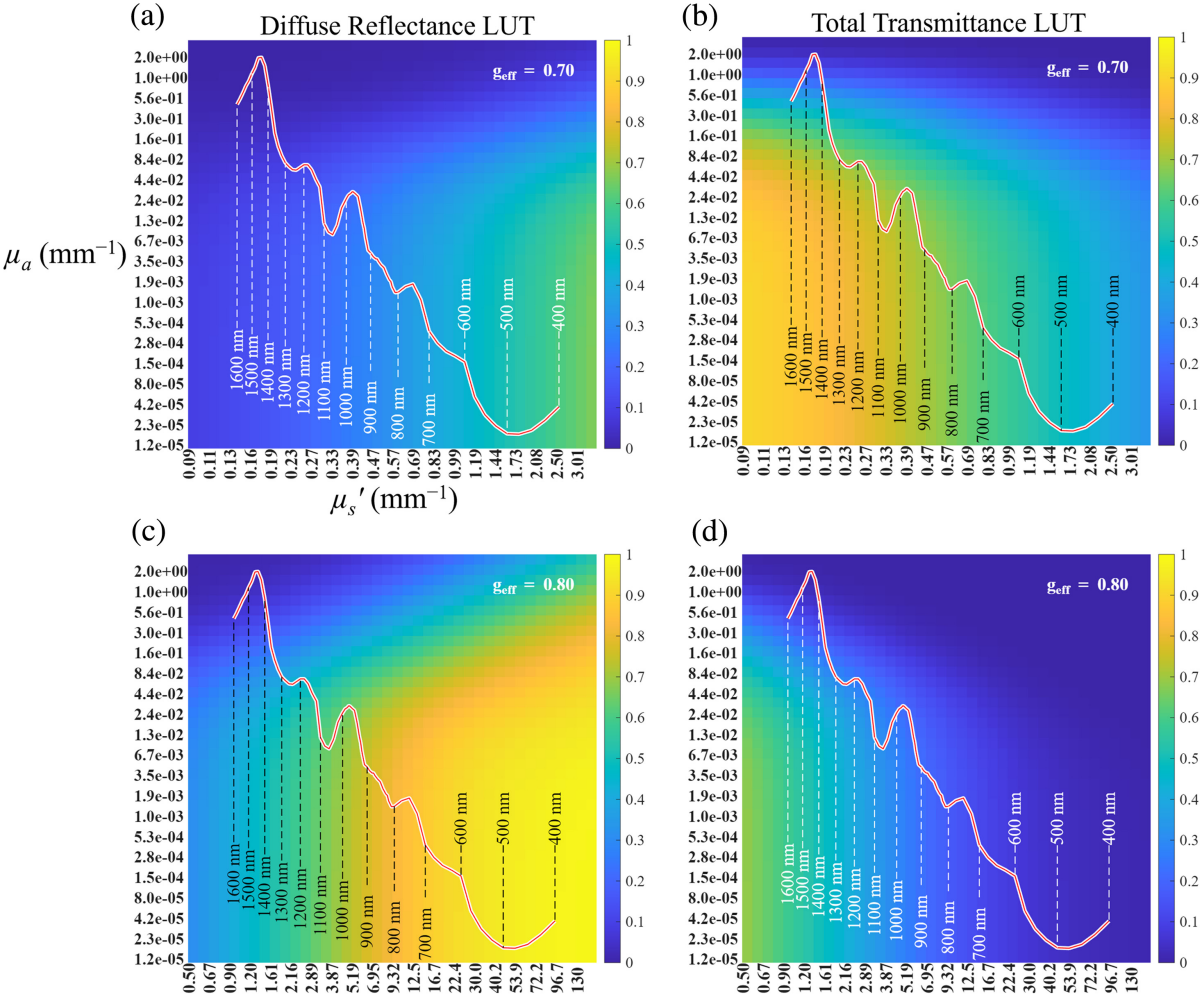
(a) Diffuse reflectance and (b) total transmittance lookup tables (LUTs) generated using MCXLAB^14^ for the transverse saline sample group. (c) Diffuse reflectance and (d) total transmittance lookup tables (LUTs) generated using MCXLAB^15^ for the longitudinal saline sample group. The overlaid red curve represents the theoretical diffuse reflectance or transmittance of tendon as a function of absorption coefficient ($\mu_{a}$) and reduced scattering coefficient ($\mu_{s}^{\prime }$) at wavelengths ranging from 400 to 1600 nm.

To model diffuse reflectance and total transmittance for tendon slices in saline, the LUTs in Figs. 9(a)–9(d) were used to determine the theoretical values for transverse and longitudinal tendon slices in saline. Figures 9(a) and 9(b) show LUTs for transverse slices generated using g=0.70, while Figs. 9(c) and 9(d) show LUTs for longitudinal slices generated using g=0.80. A sample thickness of 1.5 mm was used for all simulations. Theoretical diffuse reflectance or total transmittance as a function of $\mu_{a}$ and $\mu_{s}^{\prime }$ at wavelengths from 400 to 1600 nm are overlaid with a red curve.

**Fig. 10:**
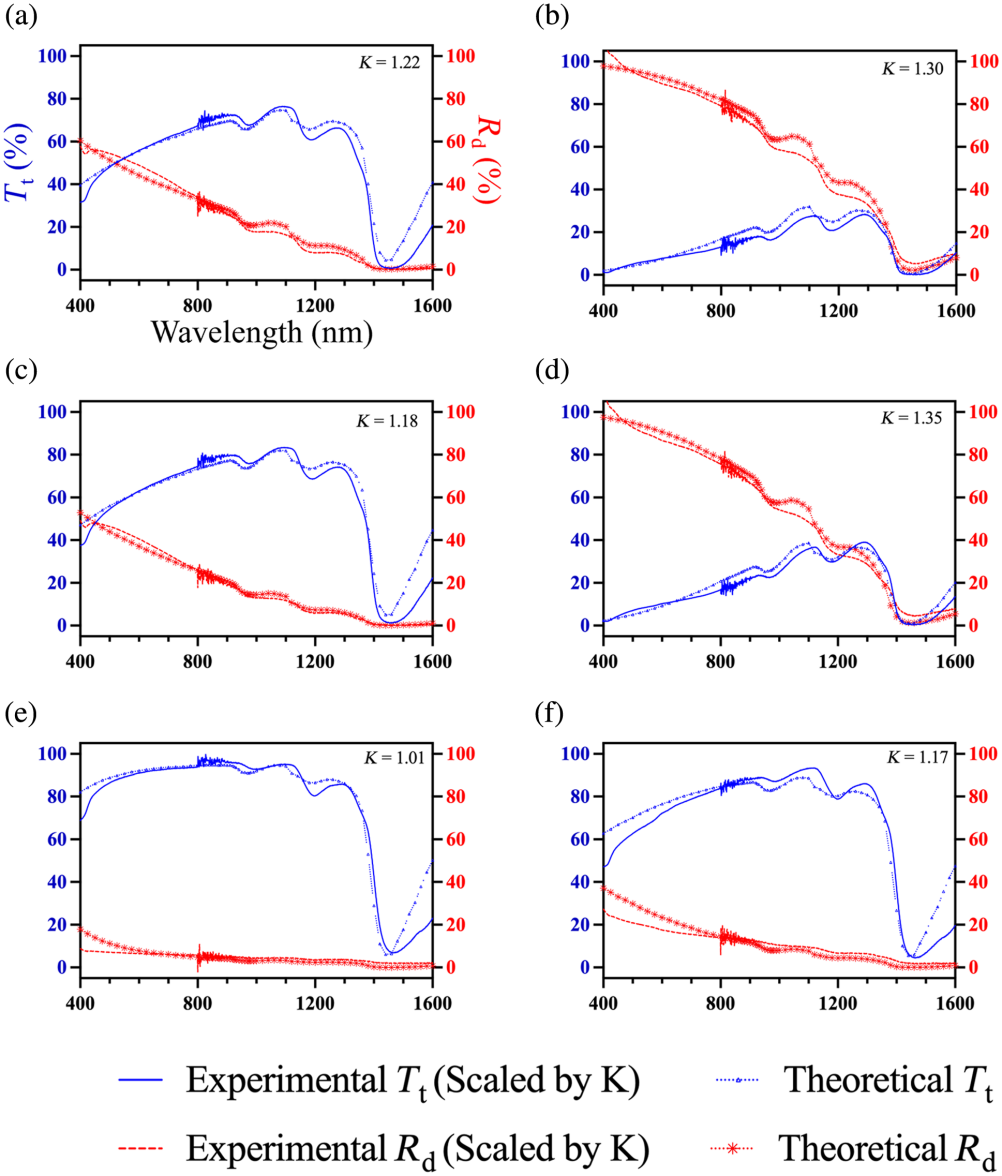
Experimental spectra and theoretical diffuse reflectance ($R_{d}$) and total transmittance ($T_{t}$) spectra for transverse and longitudinal tendon slices treated with different concentrations of glycerol. The samples were immersed in (a) and (b) saline, (c) and (d) 10% glycerol, and (e) and (f) 60% glycerol solutions. The left column (a), (c), and (e) shows results for transverse tendon slices. The right column (b), (d), and (f) shows results for longitudinal tendon slices. The experimental data are averaged and scaled by a factor K, with K values indicated in each plot.

Figure 10 presents the theoretical reflectance and transmittance spectra as functions of wavelength (dashed line), obtained from the LUTs, alongside scaled and averaged experimental data (solid line). Theoretical results matched closely with the experimental data for saline and 10% glycerol conditions. Some deviations were observed for the 60% glycerol condition. After immersion in 60% glycerol, the tendon became dehydrated and hardened, and as a result, did not sit as flush against the integrating sphere port as the other sample groups. In addition, dehydration increases the tendon’s refractive index, potentially causing some light to refract and miss the sample port. The wavelength dependence of refractive index may contribute to the greater deviation observed toward shorter wavelengths.

Parameters obtained from iterative least squares fitting of the theoretical model to the scaled and averaged experimental reflectance and transmittance spectra are listed in Tables 2 and 3 for transverse and longitudinal tendon slices, respectively. The theoretical spectra across the full wavelength range were generated by substituting these parameters into Eqs. (7) and (8), where Eq. (7) defines the water absorption spectrum scaled by tendon water content, and Eq. (8) defines the theoretical reduced scattering spectrum.

**Table 2:** Fitted model parameters for transverse tendon slices based on average spectrum of 6 specimens.

	$W_{t}$	a (${\mathrm{mm}}^{-1}$)	b	K
Tendon slice in saline	0.65	1.54	2.10	1.22
Tendon slice in 10% glycerol	0.65	1.01	2.20	1.18
Tendon slice in 60% glycerol	0.65	0.12	2.62	1.01

**Table 3:** Fitted model parameters for longitudinal tendon slices based on average spectrum of six specimens.

	$W_{t}$	a (${\mathrm{mm}}^{-1}$)	b	K
Tendon slice in saline	0.65	42.5	3.32	1.30
Tendon slice in 10% glycerol	0.65	35.5	3.53	1.35
Tendon slice in 60% glycerol	0.65	0.43	2.00	1.17

### 3.3 Reduced Scattering Coefficients

**Fig. 11:**
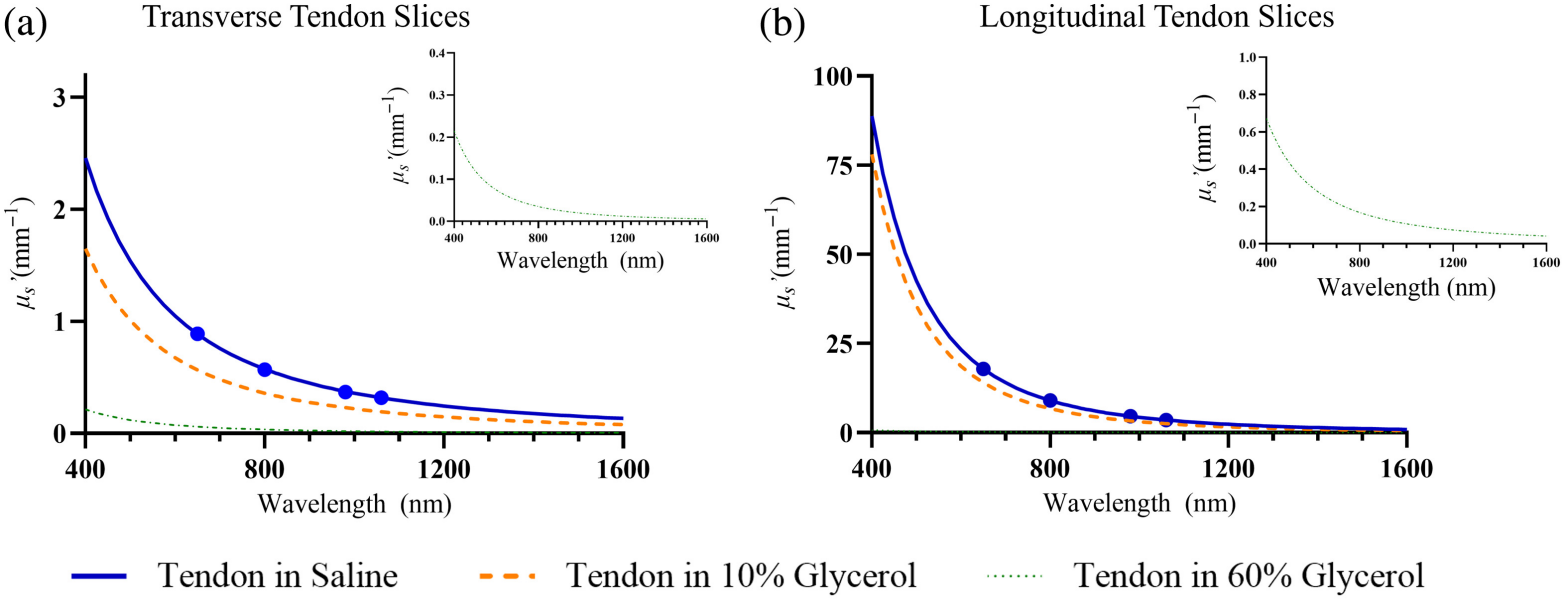
Reduced scattering coefficient ($\mu_{s}^{\prime }$) spectra for tendon slices in different solutions. (a) Transverse tendon slices and (b) longitudinal tendon slices in saline, 10% glycerol, and 60% glycerol solutions. Circles indicate the wavelengths reported in Table 4 (650, 800, 980, and 1060 nm). The insets in each panel show the reduced scattering coefficient spectra for the tendon slices in the 60% glycerol condition.

**Fig. 12:**
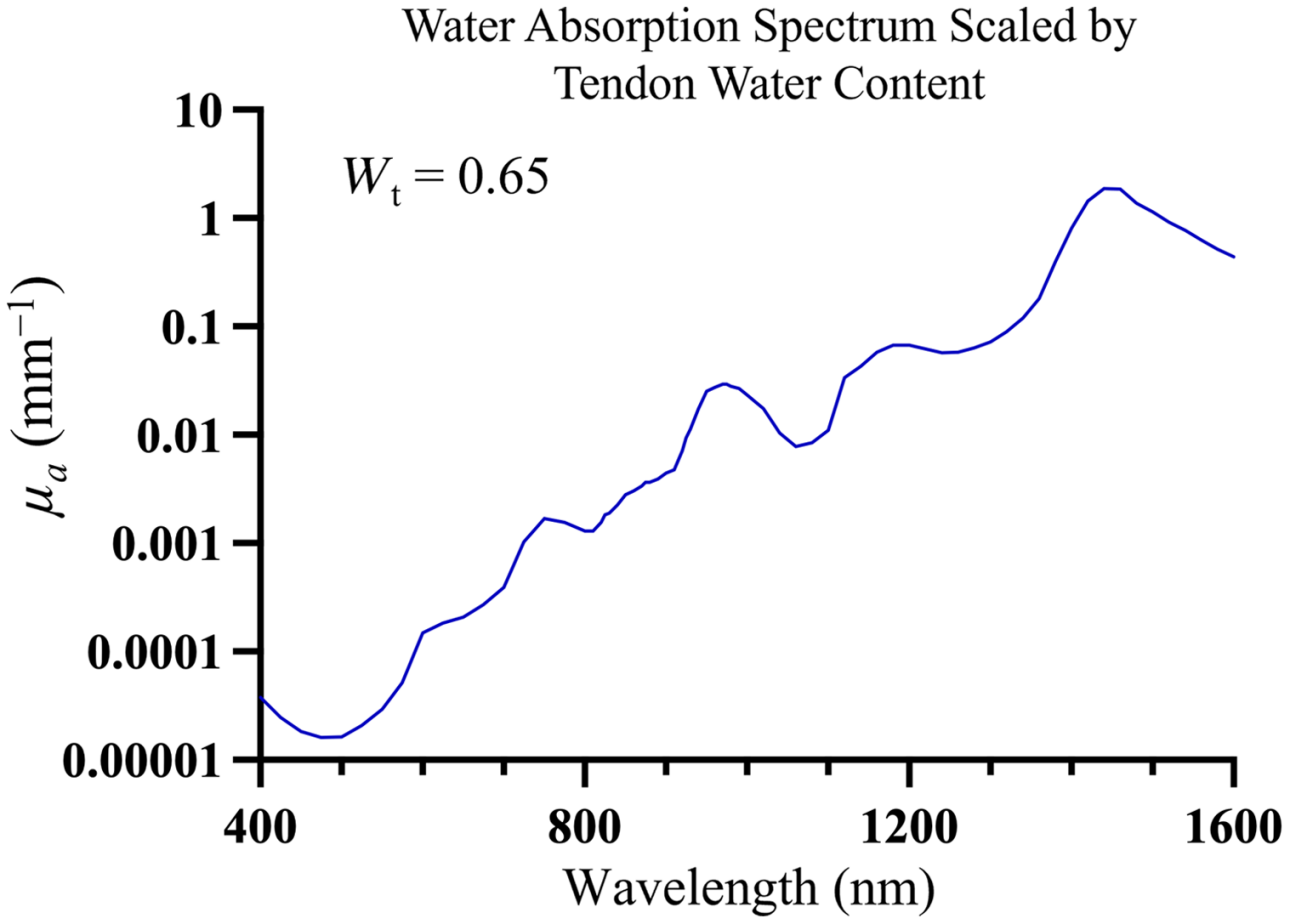
Absorption coefficient ($\mu_{a}$) spectrum of water scaled by tendon’s water content ($W_{t}$) of 0.65. This spectrum reflects the contribution of water to the tendon’s absorption properties and serves as a scaled water absorption spectrum rather than a direct tendon absorption spectrum.

The reduced scattering spectra corresponding to the theoretical reflectance and transmittance data are shown in Fig. 11. The absorption coefficient spectrum in Fig. 12 represents the water absorption spectrum^24^ scaled by the water content of tendon slices ($W_{t}=0.65$). Water content was the same across all samples (Tables 2 and 3), so the absorption spectrum is identical for all conditions.

Observations from the reduced scattering spectra reveal several key trends: both types of slices exhibit a decrease in $\mu_{s}^{\prime }$ with increasing wavelength. This trend is more marked in the visible spectrum due to the dominance of Rayleigh scattering, which has a strong inverse fourth power dependency on wavelength.^27^ In addition, Mie scattering, which is less dependent on wavelength and also present in tendons, becomes more influential at longer wavelengths.

The reduced scattering for longitudinal slices is considerably higher compared to transverse slices. Table 4 highlights the values at selected wavelengths relevant to PBMT applications. At 800 nm, the reduced scattering for longitudinal slices in saline is $9.01\;{\mathrm{mm}}^{-1}$, compared to only $0.57\;{\mathrm{mm}}^{-1}$ for transverse slices, a nearly 16-fold difference. As illustrated in Fig. 11, increasing glycerol concentration reduces $\mu_{s}^{\prime }$ in both orientations; at 800 nm, $\mu_{s}^{\prime }$ for longitudinal slices decreases from $9.01\;{\mathrm{mm}}^{-1}$ in saline to $6.76\;{\mathrm{mm}}^{-1}$ in 10% glycerol and to $0.16\;{\mathrm{mm}}^{-1}$ in 60% glycerol, while transverse slices decrease from 0.57 to $0.36\;{\mathrm{mm}}^{-1}$ and $0.04\;{\mathrm{mm}}^{-1}$, respectively.

**Table 4:** Reduced scattering coefficients ($\mu_{s}^{\prime }$) of tendon slices in saline at clinically relevant wavelengths corresponding to the highlighted points in Fig. 11. Values from the present study are reported separately for transverse and longitudinal slices of bovine tendon. Literature values from Haugen et al. (human tendon), Kienle et al. (bovine tendon), and Mosca et al. (porcine tendon) are included for comparison.

Wavelength (nm)	$\mu_{s}^{\prime }$ (${\mathrm{mm}}^{-1}$)—Parallel to fibers		$\mu_{s}^{\prime }$ (${\mathrm{mm}}^{-1}$)—Perpendicular to fibers			
	This study	Kienle et al.^20^	This study	Haugen et al.^a^ ^18^	Mosca et al.^19^	Kienle et al.^20^
650	0.89	—	17.9	12.6^b^	2.23	—
800	0.57	∼0.4 ^c^	9.01	8.50	1.07	∼2.4 ^c^
980	0.37	—	4.61	4.93	0.46	—
1060	0.32	—	3.56	3.90	0.36	—

a Fiber orientation relative to the measurement geometry was not explicitly specified by Haugen et al.; values are included for contextual comparison only and are not used to infer orientation-dependent scattering.

b Measured at 652 nm.

c Values for Kienle et al. were estimated from the published plot (Fig. 8 in Ref. 20).

### 3.4 Power Transmission Measurements

Power transmission measurements through larger tendon sections are summarized in Table 5. For tendons in saline, 17% of the incident 88 mW laser power was transmitted parallel to the fibers, whereas no light was transmitted in the perpendicular direction. The 10% glycerol condition showed transmission values similar to those in saline. In contrast, 60% glycerol increased the transmitted power to nearly 50% in both orientations, eliminating the preferential light propagation observed under saline and 10% glycerol conditions. Although the lengths of the tendon samples were not exactly equal to their widths due to the hardening effect of 60% glycerol, which made precise cutting difficult, the substantial increase in transmission from saline to 60% glycerol is consistent with the large decrease in reduced scattering observed in Fig. 11.

**Table 5:** Power measurements for tendon samples under 88 mW, 810 nm laser irradiation. Tendon dimensions for each treatment group are provided, where lengths along the long axis of the tendon were adjusted to approximately match respective width across the tendon’s short axis.

	Irradiated along the length of the fibers	Irradiated perpendicular to the fiber direction
Saline	15 mW	0 mW
	Tendon length: 13.8 mm	Tendon width: 13.8 mm
10% Glycerol	14 mW	0 mW
	Tendon length: 13.6 mm	Tendon width: 12.6 mm
60% Glycerol	40 mW	41 mW
	Tendon length: 10.9 mm	Tendon width: 8.9 mm

### 3.5 Beam Profiler Images of Large Tendon Sections

**Fig. 13:**
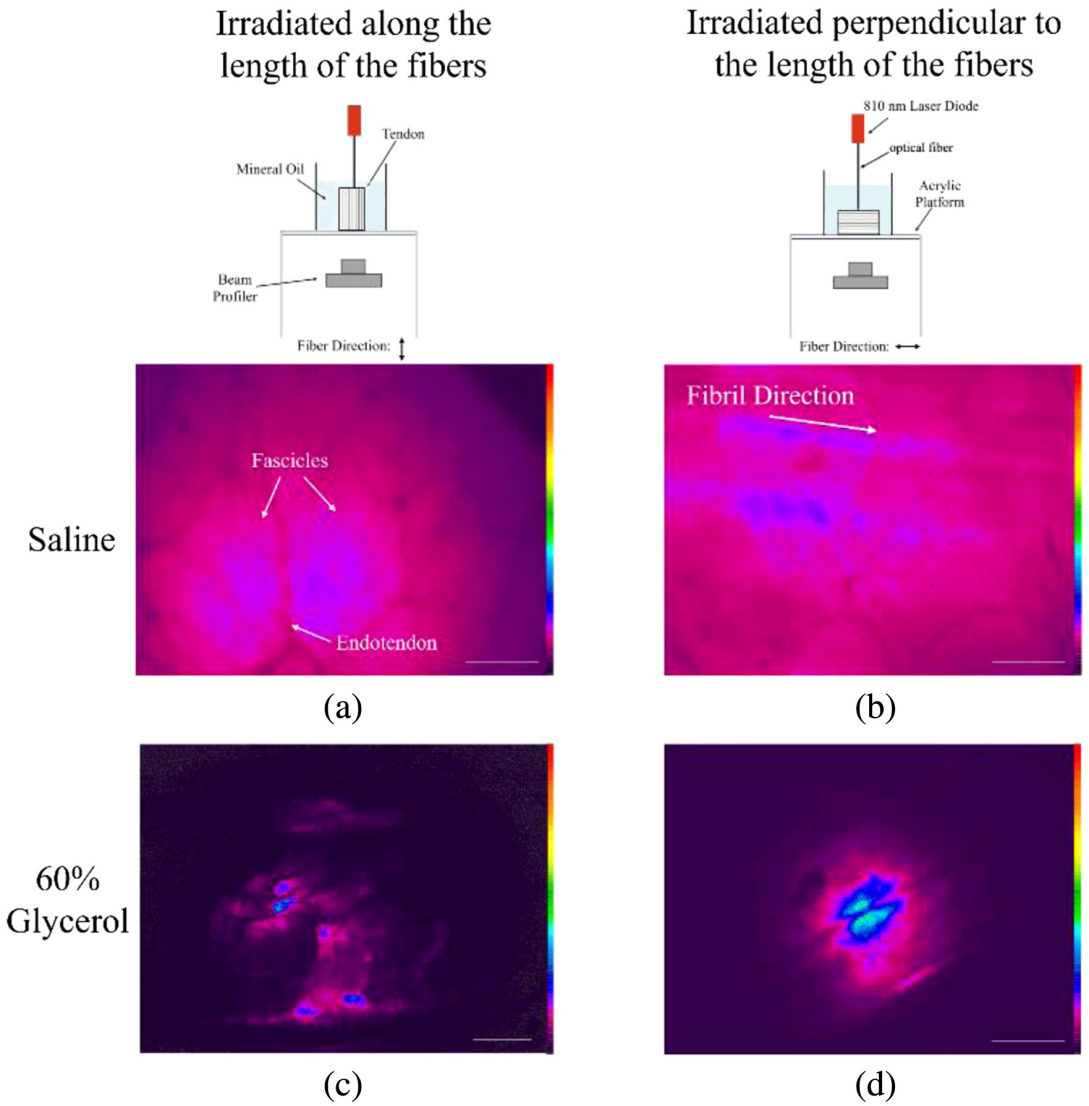
Beam profiler images of tendon samples in saline and 60% glycerol, placed in mineral oil. Panels (a) and (c) show samples irradiated parallel to the fiber direction, and panels (b) and (d) show samples irradiated perpendicular to the fiber direction, both imaged from below. All images share the same color bar, though the colors do not represent actual power differences. Scale bar = 1 mm.

Figure 13 presents beam profiler images of tendon samples immersed in saline and 60% glycerol, placed in a holder filled with mineral oil; some images contain visible air bubbles within the oil. To prevent camera sensor saturation, several images were captured using an additional neutral density filter. Therefore, although all images use the same color bar, the colors, which represent pixel intensities, do not accurately reflect the actual power differences between the samples. When irradiated parallel to the fibers, fascicle structures appear brighter than the surrounding endotendon in saline [Fig. 13(a)], indicating that light propagation is influenced by the tendon’s internal structure along the fiber direction. Upon immersion in 60% glycerol, this structured appearance diminishes, with light appearing more concentrated in several disconnected areas rather than evenly distributed across the tendon surface [Fig. 13(c)].

When the tendon is irradiated perpendicular to the fibers, light still preferentially travels along the fiber length, creating elongated patterns [Fig. 13(b)]. This suggests that light preferentially propagates along the fiber axis even when incident perpendicular to it. After immersion in 60% glycerol, much of this structured light propagation is lost, leaving only a slightly elongated spot [Fig. 13(d)].

Small variations in tissue thickness could contribute to localized intensity differences in these images. However, the bright regions in Fig. 13(a) consistently align with fascicle orientation rather than appearing in irregular areas.

## 4 Discussion

This study demonstrates that tendons are optically highly anisotropic, exhibiting differences in reduced scattering coefficients ($\mu_{s}^{\prime }$) of at least an order of magnitude when measured parallel (transverse slices) versus perpendicular (longitudinal slices) to the fiber direction. Specifically, at clinically relevant wavelengths such as 650, 800, and 980 nm, the differences between fiber orientations were 20-fold, 16-fold, and 13-fold, respectively. This work explores how fiber orientation affects $\mu_{s}^{\prime }$ across a broad spectral range, extending prior studies that have examined fiber orientation and wavelength as independent factors.^18^^–^^20^

At 800 nm, the reduced scattering coefficient was $9.01\;{\mathrm{mm}}^{-1}$ for longitudinal slices and $0.57\;{\mathrm{mm}}^{-1}$ for transverse slices. Haugen et al. reported $∼8.5\;{\mathrm{mm}}^{-1}$ in human tendon at 800 nm using an integrating sphere method. Mosca et al. measured $∼1.07\;{\mathrm{mm}}^{-1}$ in fresh porcine tendon using time-domain diffuse optical spectroscopy (TD-DOS) through a 1-cm multilayer slab. Kienle et al. reported reduced scattering coefficients at 800 nm of $∼0.4\;{\mathrm{mm}}^{-1}$ (parallel to fibers) and $2.4\;{\mathrm{mm}}^{-1}$ (perpendicular to fibers). Their parallel measurement is comparable in magnitude to the transverse value reported here, while their perpendicular value remains substantially lower than the longitudinal measurement.^20^ Differences in reported values across studies likely reflect variations in species, sample geometry, fiber orientation, and measurement methodology rather than disagreement in underlying tissue behavior.

The measurements presented here align well with the theoretical models [Figs. 10(a) and 10(b)]. However, the measured diffuse reflectance and total transmittance did not sum to 100% at wavelengths with negligible water absorption (e.g., 500 nm). A constant scaling factor K was therefore applied so the measurements would sum closer to 100%. This correction assumes that diffuse reflectance and transmittance were underestimated by the same proportion, which may not be strictly accurate. In a single-port integrating sphere setup, transmitted photons pass through the full sample thickness and can spread laterally, allowing some to escape the tissue before entering the sphere. Diffuse reflectance, which is measured from the surface of the sample that faces into the sphere, does not depend on transport through the thickness of the sample. If transmission is underestimated to a greater extent than diffuse reflectance, applying the same scaling factor to both measurements can make the recovered $\mu_{s}^{\prime }$ appear higher than the true value. For similar reasons, the fitted water content may also be slightly overestimated if part of the loss is attributed to absorption. To prevent unphysical values during fitting, the upper bound on water content was constrained during optimization. The bovine tendons used in this work were obtained from a butcher shop, so the specific tendon type, animal age, and any prior freeze–thaw cycles are not known. Although these factors may shift the absolute values,^28^ they do not change the primary result of this work: reduced scattering is substantially higher for light incident perpendicular to the fibers than parallel to them.

The striking difference in reduced scattering coefficients parallel versus perpendicular to the fibers is consistent with predictions derived from solutions to Maxwell’s equations in cylindrical coordinates describing scattering by an infinitely long cylinder.^20^^,^^27^^,^^29^^,^^30^ These solutions show increased scattering efficiency when the angle of incident light is perpendicular to the cylinder’s long axis (incident angle = 90 deg), and decreased efficiency when the angle is parallel to the cylinder axis (incident angle = 0 deg). Furthermore, at oblique incident angles, light travels in a cone that narrows at smaller angles.^31^ A Monte Carlo model by Kienle et al., who applied these concepts to explain multiple scattering by a distribution of cylinders representing dentin tubules, showed that the photon path follows the direction of the tubules.^32^ This behavior may also apply to tendon, as higher light intensity is observed along the fibers in beam profiler images (Fig. 13).

The anisotropy coefficients obtained from goniometer measurements in the present work reflect the forward scattering region (±50  deg) and should be interpreted accordingly. True anisotropy g is strongly influenced by the backscattered light, which is missing from the data. Therefore, the reported value, referred to here as $g_{\mathrm{eff}}$, might not accurately represent the true value. However, because light penetration in tendon is governed primarily by forward-directed scattering, $g_{\mathrm{eff}}$ remains relevant to the transport behavior examined in this study.

Under saline conditions (Fig. 6), the goniometer results showed higher forward-directed scattering for longitudinal slices ($g_{1,\mathrm{eff}}=0.94$) compared to transverse slices ($g_{1,\mathrm{eff}}=0.90$). When light is incident perpendicular to the fiber axis (longitudinal slices), it likely interacts with the collagen fibers across a larger cross section, which may increase forward scattering. In the transverse orientation, where light travels along the fiber axis, the scatterers are smaller and more isotropic, producing a broader angular distribution. Small holes in some samples, as well as slight compression from mounting the tissue between the slide and the prism during measurements, may also have altered fiber alignment and contributed to variability in the measured angular distributions.

The anisotropy (g) and the reduced scattering coefficient $\mu_{s}^{\prime }$ [see Eq. (1)] can be related through Eq. (9) to estimate the scattering coefficient $\mu_{s}$.

$$\mu_{s}=\frac{\mu_{s}^{\prime }}{1-g}.$$ (9)

From this, the mean free path (mfp) can be calculated [Eq. (10)], which helps determine whether the 20  μm slice thickness at 633 nm was thin enough for the goniometer measurements to represent single scattering.

$$\mathrm{mfp}=\frac{1}{\mu_{s}}.$$ (10)

Table 6 shows $g_{\mathrm{eff}}$, $\mu_{s}^{\prime }$, and the mean free path at 633 nm for the transverse and longitudinal slices.

**Table 6:** Measured $g_{\mathrm{eff}}$ and $\mu_{s}^{\prime }$ values for transverse and longitudinal saline-soaked tendon slices at 633 nm, and the corresponding mean free path (mfp).

Slice	$\mu_{s}^{\prime }$ (${\mathrm{mm}}^{-1}$)	$g_{\mathrm{eff}}$	$\mu_{s}$ (${\mathrm{mm}}^{-1}$)	mfp (mm)
Transverse	0.94	0.70	3.14	0.318
Longitudinal	19.6	0.80	98.0	0.010

The results show that the transverse mean free path (mfp=318  μm) is much greater than the 20  μm thickness of the slices used in the goniometer measurements. Therefore, the angular scattering measured for the transverse slices is expected to represent the single scattering function. In contrast, the longitudinal mean free path (mfp≈10  μm) indicates that a 20  μm slice corresponds to roughly two mean free paths, so the measured angular distribution is not strictly representative of single scattering.

This distinction, however, has only a modest effect on the reduced scattering coefficients obtained from the LUT analysis. Diffuse reflectance and transmittance from a 1.5-mm-thick slab, which is several mean free paths in thickness, depend predominantly on the reduced scattering coefficient $\mu_{s}^{\prime }$ rather than on $\mu_{s}$ and g individually. After many scattering events, the influence of the single-scattering phase function becomes small. Consistent with this, the recovered $\mu_{s}^{\prime }$ showed only small shifts when LUTs generated with different g inputs were tested. Thus, this deviation from single scattering in the longitudinal slices is unlikely to meaningfully alter the recovered $\mu_{s}^{\prime }$.

Table 5 shows how tendon structure affects light transmission in larger, approximately centimeter-long sections. At 810 nm, 17% of the incident 88 mW laser power was transmitted parallel to the fibers, whereas no measurable transmission occurred in the perpendicular direction. This finding has broader implications for other clinically relevant PBMT wavelengths. At 1064 nm, a commonly used PBMT wavelength, scattering is even lower than at 810 nm. While it exhibits higher water absorption than 810 nm, it features a dip in water absorption compared to nearby wavelengths. These optical properties suggest that 1064 nm may allow greater light propagation to the tendon when light is applied externally, particularly in individuals with darker skin tones, whereas wavelengths of higher melanin absorption, such as 810 nm, may reduce light penetration to the tendon.

A wavelength of 980 nm is also clinically relevant for PBMT, though its water absorption is relatively high. Despite this, it has been shown to achieve biological effects at lower fluences compared to other wavelengths. For instance, 980 nm primarily targets temperature-sensitive calcium ion channels, such as TRP channels, whereas 810 nm primarily stimulates mitochondrial pathways. The fluence required for these effects differs significantly. Wang et al. found that adipose-derived stem cells responded to 810 nm with peak proliferation at a dose 10 to 100 times greater than that required for 980 nm.^33^ Incorporating wavelength optimization alongside the use of optical clearing agents such as glycerol could further improve PBMT outcomes in tendons.

Treatment of tendons with 60% glycerol significantly increased light penetration and reduced the directional preference of light propagation along the fiber direction, making the tendons nearly transparent in both fiber orientations (Table 5). This process caused the tendons to harden and decrease in width, suggesting that 60% glycerol dehydrates the tendon by drawing out water more quickly than glycerol diffuses into the tissue.

Both 10% and 60% glycerol lowered the reduced scattering coefficient $\mu_{s}^{\prime }$, with the effect being modest at 10% and pronounced at 60% (Fig. 11). The reduced scattering coefficient $\mu_{s}^{\prime }$ is influenced by both the scattering coefficient $\mu_{s}$ and the anisotropy factor g [see Eq. (1)]. Goniometer measurements showed increasingly forward-directed scattering after glycerol treatment. This suggests that dehydration causes fibrils within the tendon to pack more closely together, resulting in constructive interference of scattered light and more forward-directed scattering for both fiber orientations.^34^ According to Mie theory, larger scatterers exhibit a higher anisotropy factor.^35^^,^^36^ Therefore, it is reasonable to infer that the tighter packing of fibrils forms larger scatterers, which increases g and subsequently lowers $\mu_{s}^{\prime }$.

A change in the scattering coefficient $\mu_{s}$ can also lower the reduced scattering coefficient $\mu_{s}^{\prime }$. The refractive index of 60% glycerol (∼1.42)^37^ more closely matches that of collagen fibers in tendons, which exhibit birefringence with average refractive indices of 1.36 (range: 1.32 to 1.45) in the axial orientation and 1.49 (range: 1.40 to 1.62) in the radial orientation.^38^^,^^39^ A closer match in refractive index along either orientation reduces light scattering in that direction, leading to a lower scattering coefficient ($\mu_{s}$). In addition, as water is drawn out, the concentration of proteoglycans in the interfibrillar space may increase, raising the refractive index in these regions. These mechanisms have been proposed as contributing factors to the optical clearing effects with anhydrous glycerol in other tissues.^21^

While 10% glycerol had an obvious effect on the optical properties of thinner 1.5 mm and 20  μm tendon samples, it did not increase light penetration in larger tendon sections (Table 5), suggesting that its osmotic concentration was insufficient. Since 60% glycerol, although effective, may not be safe for clinical use, further research into glycerol concentrations that avoid systemic and local adverse effects is necessary for enhancing light propagation into tendons, given that the specific clinical application is carefully considered. Beam profiler images indicate that optical clearing with 60% glycerol results in localized irradiation rather than a spread-out effect (Fig. 13). Lower concentrations might offer a more balanced distribution between deeper penetration and broader coverage within the tendon, which could be preferred in certain clinical scenarios.

For practical implementation, any optical clearing method would need to be feasible in a clinical setting. In this study, samples were immersed in their solutions for 48 h before measurements to ensure adequate penetration. However, *in vivo* optical clearing with glycerol has been shown to occur much more rapidly and is temporary. For example, skin has been shown to clear within minutes and revert to baseline within 30 min after injection of 20% or 30% glycerol solutions.^40^ Whether a similar rapid effect would occur in tendon is unknown, and the response to injection cannot be assumed to match the behavior of tissue that has been soaked *ex vivo*.

Returning to the original question, “Does delivery of light along the tendon fibers improve the penetration of treatment light for PBMT, in comparison to delivery across the tendon fibers? the power transmission experiment of Fig. 4 was simulated using Monte Carlo simulations with the parameters from Tables 2 and 3, which yielded optical parameters at 810 nm wavelength. For transverse section soaked in saline: $\mu_{a}=0.0013/\mathrm{mm}$, $\mu_{s}^{\prime }=0.56/\mathrm{mm}$, g=0.70, and $\mu_{s}=1.87/\mathrm{mm}$. For the longitudinal section soaked in saline: $\mu_{a}=0.0013/\mathrm{mm}$, $\mu_{s}^{\prime }=8.6/\mathrm{mm}$, g=0.80, and $\mu_{s}=43.4/\mathrm{mm}$. The tendon was represented as a 13.8  mm×13.8  mm×50  mm volume. A ∼200-μm-diameter optical fiber was placed in contact with the 13.8  mm×13.8  mm face, directing light along the 50 mm length to replicate the experimental geometry. Light on the lateral sides of the tendon slice was allowed to escape, mimicking escape into surrounding tissue, likely muscle, which would have lower scattering and higher absorption due to blood perfusion.

**Fig. 14:**
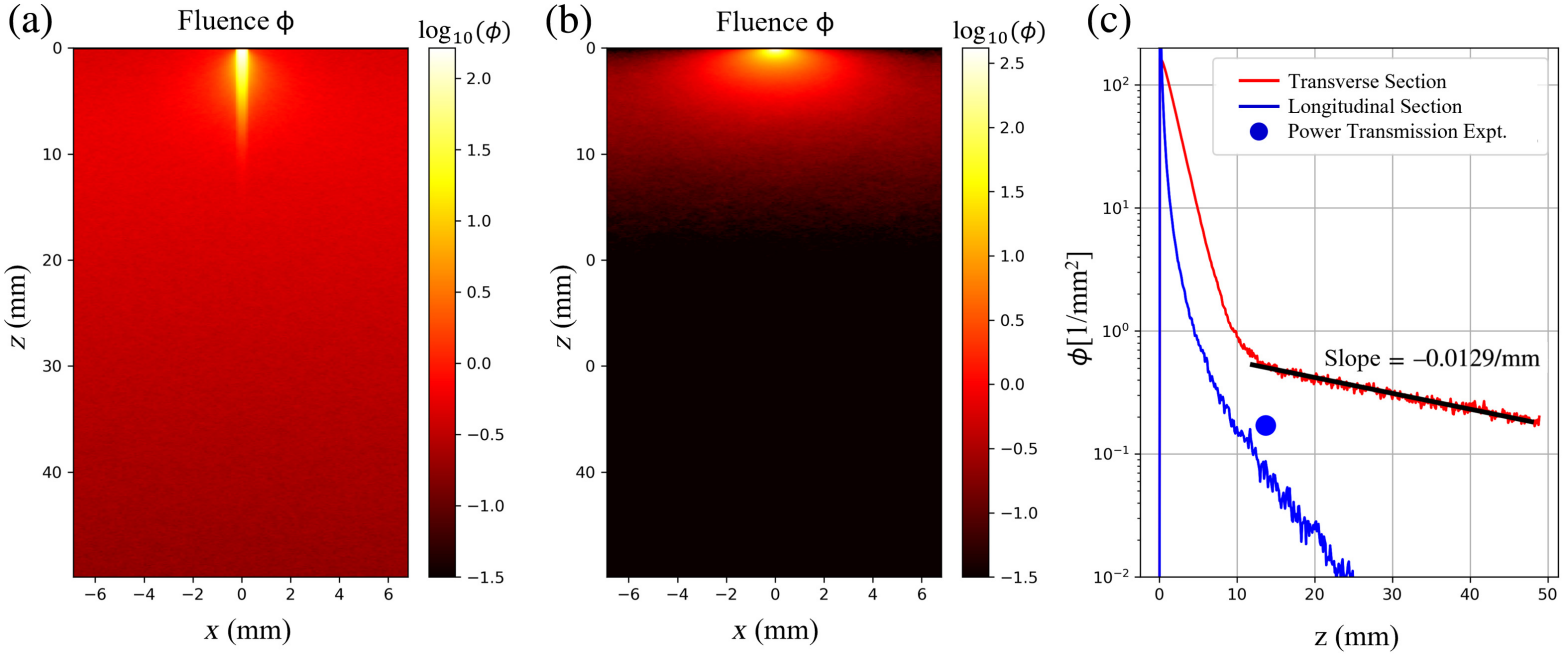
(a) Photon fluence ϕ [$1/{\mathrm{mm}}^{2}$] versus x, z for the transverse section. (b) Photon fluence ϕ [$1/{\mathrm{mm}}^{2}$] versus x, z for the longitudinal section. (c) Depth profiles ϕ(z) at x=0, y=0 for both transverse and longitudinal sections. The blue circle indicates the detected transmission in the experiment of Fig. 4.

Figure 14 shows the results. There is significant penetration of the 810-nm treatment light in the tendon when light is delivered parallel to the tendon fibers. Sharp attenuation occurs when light is delivered perpendicular to the tendon fibers. The blue circle represents the result of the power transmission experiment (see Table 5): $T=\frac{15\text{ mWdetected}}{88\text{ mWdelivered}}=0.17$. The key result is the gradual slope of attenuation ($\mu_{\mathrm{eff}}=0.020/\mathrm{mm}$) for the transverse section, enabling treatment light to penetrate up to 5 cm. The tendon was assumed not to have any blood content, which explains the low attenuation. In contrast, delivery across the tendon fibers (the longitudinal section) is sharply attenuated by the much stronger scattering. The experimentally measured transmission (blue circle) does not exactly coincide with the simulated transverse attenuation curve (red), but it is in the neighborhood. The details of the boundary conditions and cross-sectional shape of the tendon were not fully addressed in the simulations. Nevertheless, the simulations illustrate the ability of an 810-nm treatment light to achieve significant penetration when delivered parallel to the tendon fibers.

The axial propagation observed here motivates future investigation into whether the tendon could function as a conduit for light transmission along its long axis, potentially extending delivery toward adjacent muscle regions commonly targeted in PBMT. The branching architecture of certain tendons could further enable the distribution of light to multiple connected muscle groups, although this remains to be explored. Interstitial light delivery approaches, where a fiber optic is introduced via needle insertion,^41^ reduce attenuation by overlying tissues. In combination with the greater axial propagation observed here, interstitial delivery may allow light to reach deeper regions than external irradiation.

An additional area for future study concerns the optical mechanisms that may contribute to the greater propagation observed along the tendon fiber direction. Beam profiler images (Fig. 13) showed higher intensity within fascicles relative to the surrounding endotendon, and individual fibers exhibited greater intensity than the interfibrillar matrix. Although these patterns may be explained by multiple scattering, light guiding or total internal reflection (TIR) may also contribute. The potential influence of refractive index differences, particularly in intrasynovial tendons encased by synovial sheaths, warrants further investigation, as refractive index mismatch may contribute to guided propagation along the fiber axis. Introducing a lower refractive index material, such as air, into the synovial sheath could further enhance light transfer along the tendon axis. Investigating these mechanisms may provide new insights into optimizing light delivery for PBMT and related applications.

## 5 Conclusion

Fiber orientation significantly affects light transport properties across a broad wavelength range, with differences exceeding an order of magnitude at wavelengths commonly used in PBMT. Monte Carlo simulations using the measured optical coefficients demonstrated how these orientation-dependent differences influence light propagation in bulk tissue, illustrating substantially greater penetration of 810-nm treatment light delivered parallel to tendon fibers compared to perpendicular delivery.

Glycerol reduces overall scattering and makes scattering more forward directed, increasing light penetration in both fiber orientations. However, further research is required to determine clinically safe and effective concentrations. With expanded investigation across species and tissue states, and continued evaluation of measurement methodology, the orientation-dependent transport differences identified here may ultimately inform future clinical dosimetry studies.

## Data Availability

The data presented in this article is publicly available in GitHub at https://github.com/alexaalisa/Structure-of-tendon-causes-highly-optical-properties-and-transport/tree/main.
